# In Vitro and In Vivo Biosafety Analysis of Resorbable Polyglycolic Acid-Polylactic Acid Block Copolymer Composites for Spinal Fixation

**DOI:** 10.3390/polym13010029

**Published:** 2020-12-23

**Authors:** Seung Kyun Yoon, Jin Ho Yang, Hyun Tae Lim, Young-Wook Chang, Muhammad Ayyoob, Xin Yang, Young Jun Kim, Han-Seung Ko, Jae Young Jho, Dong June Chung

**Affiliations:** 1 School of Chemical Engineering, Sungkyunkwan University, 2066 Seobu-Ro, Jangan-Gu, Suwon, Gyeonggi 16419, Korea; yyskyoon@skku.edu (S.K.Y.); ch_ayyoob_kamboh@live.com (M.A.); xin12.25@hotmail.com (X.Y.); youngkim@skku.edu (Y.J.K.); 2 Department of Materials & Chemical Engineering, Hanyang University, 55 Hanyangdaehak-Ro, Sangnok-Gu, Ansan, Gyeonggi 15588, Korea; sheep@hanyang.ac.kr (J.H.Y.); hyuntaet3t@hanyang.ac.kr (H.T.L.); ywchang@hanyang.ac.kr (Y.-W.C.); 3 School of Chemical and Biological Engineering, Seoul National University, 1 Gwanak-Ro, Gwanak-Gu, Seoul 08826, Korea; khs88@snu.ac.kr (H.-S.K.); jyjho@snu.ac.kr (J.Y.J.)

**Keywords:** PGA–PLA block copolymer, bioresorbable, composite, in vivo degradation test, MTT assay

## Abstract

Herein, spinal fixation implants were constructed using degradable polymeric materials such as PGA–PLA block copolymers (poly(glycolic acid-b-lactic acid)). These materials were reinforced by blending with HA-g-PLA (hydroxyapatite-graft-poly lactic acid) and PGA fiber before being tested to confirm its biocompatibility via in vitro (MTT assay) and in vivo animal experiments (i.e., skin sensitization, intradermal intracutaneous reaction, and in vivo degradation tests). Every specimen exhibited suitable biocompatibility and biodegradability for use as resorbable spinal fixation materials.

## 1. Introduction

Nowadays, resorbable materials are often employed to construct spinal fixation implants commonly used to restore damaged bone or bone tissue during orthopedic surgery. This type of surgery is particularly suited for elderly patients as it eliminates the need for a second surgery for removing the implant. Internal fixation implants are employed for spinal injury recovery as they exhibit improved mechanical properties and the reduced risk of implant rejection by the bone and body. Implants, such as screws, plates, pins, and suture anchors, mainly consist of stainless steel, titanium, or their respective alloys. Unfortunately, the use of metallic implants for bone fixation has intrinsic risks such as the stress-shielding phenomenon often seen in clinical images, excessive pain, and local irritation [1,2,3]. Additionally, metallic plate screws are known to damage the surrounding bone tissues, contributing to osteoporosis [4]. For these reasons, secondary surgery is necessary to remove the metallic implants after the bone or bone tissue has recovered [2]. The shortcomings of the metallic fixation implant system can be overcome by using bioresorbable and biodegradable polymeric materials, as these materials eliminate the need for a second surgery, exhibit radiolucency, are corrosion resistant, and prevent the accumulation of metal ion in tissues; additionally, the use of these materials is associated with less pain and promotes the stress-shielding effect [5,6,7]. Although these implants are often manufactured from polylactic acid (PLA), polyglycolic acid (PGA), and their random copolymer (PLGA), their biodegradability due to the presence of non-resorbable moieties is still unknown, particularly as it relates to the effects exerted on the strength-enhancing self-reinforcing traits of these materials [8,9]. Meanwhile, the PGA–PLA block copolymer facilitates control of the rate of degradation, produces fewer byproducts, and exhibits higher tensile strength when compared to its PGA–PLA random copolymer counterpart [10,11,12]. Additionally, the extent of degradation in the implanted materials depends on a blend of complex factors, including contact with body fluids, prevailing body temperature, tissue environment, the molecular weight of the implant, crystallinity, and the geometry of the implants [13].

In this study, two types of highly efficient PGA–PLA block copolymers and their composites were synthesized for use in bone fixation devices and reinforced by blending with HA-g-PLA (hydroxyapatite-graft-PLA) fillers and PGA fiber. Subsequent characterization was conducted via in vitro (MTT assay) and in vivo biosafety analyses (i.e., skin sensitization, intradermal intracutaneous reaction, and in vivo biodegradation tests). Biodegradation tests were conducted using animal models via histochemical analysis.

## 2. Materials and Experimental

### 2.1. Materials

#### 2.1.1. PGA–PLGA Multiblock Copolymer (PGA–b-PLA)

L-lactide (LA, Supomer-L^®^) and glycolide (GL, Supomer-GLD^®^), which were used as the monomers for ring-opening polymerization, were purchased from Medichem Co., Ltd. (Gongju, Korea). 1,4-Butanediol (BD), tin(II) 2-ethylhexanoate (Sn(Oct)_2_), and 1,6-hexamethylene diisocyanate (HDI), which were used as the initiator, catalyst, and chain extension agent, respectively, were purchased from Sigma–Aldrich Korea (Seoul, Korea). All reagents were used without further purification.

#### 2.1.2. PGA–PLA Triblock Copolymer (b-PLLGA)

LA, GL (>99% purity), stannous octoate, and BD were purchased from Sigma–Aldrich Korea. 1,1,1,3,3,3-Hexafluoro-2-propanol (HFIP, purity 99%) was acquired from Fluorochem Ltd. (Derbyshire, UK) and was used as received without further purification. Toluene, trifluoroacetic acid, and chloroform (Sigma-Aldrich Korea, Seoul, Korea) were vacuum distilled before use and stored over activated molecular sieves at room temperature.

#### 2.1.3. PGA–PLA Block Copolymer Composites

PGA fiber was supplied by Meta Biomed Co., Ltd. (Cheongju, Korea). Hydroxyapatite (HA) was purchased from Nanjing Emperor Nano Materials Co., Ltd. (Beijing, China) under the trade name of NHAP04. LA was purchased from Tokyo Chemical Industry Co., Ltd. (Tokyo, Japan). Sn(Oct)_2_ was purchased from Sigma-Aldrich Korea (Seoul, Korea).

#### 2.1.4. In Vitro Cytotoxicity Test

Human osteoblast cells (HOb) were obtained from Cell Applications Inc. (San Diego, CA, USA). HOb (Cat. 406K-05a) and HOb growth medium (Cat. 417-500) containing 10% of FBS, penicillin (100 μg/mL), streptomycin sulfate (100 μg/mL), and amphotericin B (250 ng/mL) were obtained from Cell Applications Inc. (San Diego, CA, USA). 3-(4,5-Dimethylthiazolyl-2)-2,5-diphenyltetrazolium bromide (MTT) and dimethyl sulfoxide (DMSO) were purchased from Sigma-Aldrich Korea (Yongin, Korea).

### 2.2. Experimental

#### 2.2.1. Synthesis of Poly(L-lactic acid)-b-poly(glycolic acid) Multiblock Copolymer (PGA–b-PLA)

**Figure 1:**
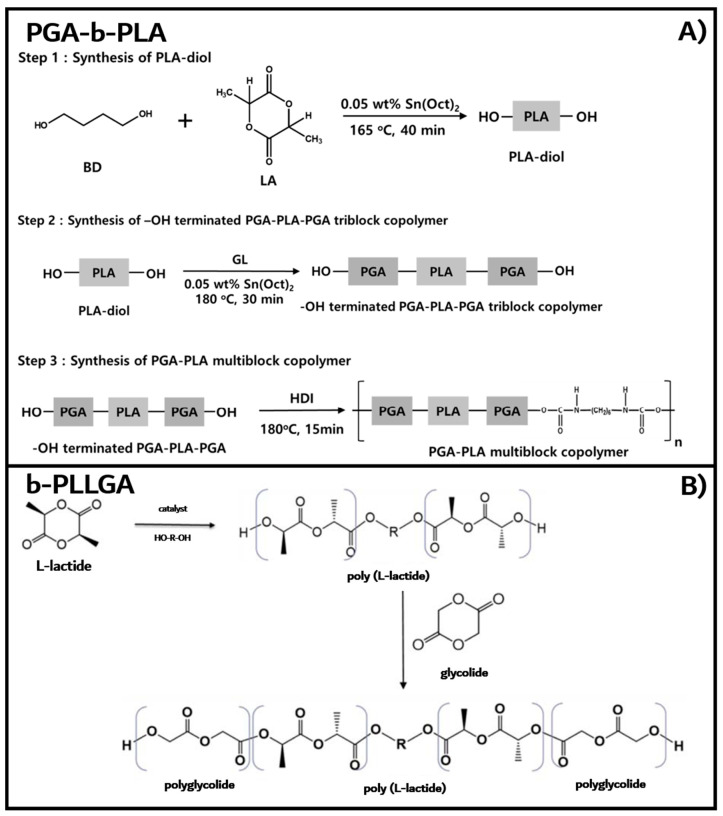
Synthesis of the PGA–PLA multiblock (poly(glycolic acid-b-lactic acid)—PGA–b-PLA) (**A**) and PGA–PLA triblock (b-PLLGA) copolymers. (**B**).

LA and GL were used for copolymerization after drying for 24 h in a vacuum oven. The PGA–b-PLA multiblock copolymers were synthesized in three steps, as shown in Figure 1A. The oligomeric PLA diol was first synthesized via the ring-opening polymerization of LA at 165 °C for 40 min under nitrogen using BD and Sn(Oct)_2_ as the initiator and the catalyst, respectively [14] The obtained PLA oligomer was dissolved in chloroform and precipitated in excess methanol to remove any unreacted monomer. Next, the PGA–PLA–PGA triblock copolymer with two hydroxyl groups in both ends was synthesized via the ring-opening polymerization of GA using the oligomeric PLA diol at 180 °C for 30 min under nitrogen, followed by the final extension step of the PGA–PLA–PGA triblock chains using HDI via urethane bond formation [15].

#### 2.2.2. Synthesis of Poly(L-lactic acid)–b-poly(glycolic acid) Triblock Copolymers (b-PLLGA)

The method of synthesis of b-PLLGA, as previously reported [16], is illustrated in Figure 1B. Briefly, LA and GL were recrystallized twice and dried under vacuum at 80 °C for 24 h before being stored at room temperature over a desiccant. A predetermined amount of LA, the initiator (BD), and the catalyst (Sn(Oct)_2_) were charged using a glovebox in a three-necked round-bottomed flask. The reaction mixture in the flask was vacuum-flushed and sealed with dry nitrogen using a Schlenk line. The sealed flask was then immersed in a hot oil bath at a preset temperature of 120 °C for 6 to 8 h. After a prescheduled reaction time, the temperature was raised to 175–185 °C depending on the monomer used in the reaction to maintain the initiator ratio and kept at this temperature until the reaction was complete. The flask was unsealed by purging with dry nitrogen, and a predetermined amount of GL (i.e., a LA:GA ratio of 70:30) was added to the reaction mixture under dry nitrogen flow depending on the molar ratio. The reaction mixture was mixed thoroughly via vigorous stirring for 30 min, and the temperature was then lowered to 150–160 °C while the reaction was continued for another 12 h. The reaction mixture was cooled to room temperature and poured in HFIP. The polymer was precipitated in chilled methanol, and the obtained white solid polymer was dried at room temperature under vacuum for 48 h and then subjected to treatment at 80 °C for 2 h to remove any residual solvent.

#### 2.2.3. PGA–PLA Block Copolymer Composites

The polymers (i.e., PGA–b-PLA and b-PLLGA) were dried at 50 °C for 24 h in a vacuum oven prior to experimentation. HA was grafted by PLA (HA-g-PLA) using Sn(Oct)_2_ to enhance compatibility with the PGA–PLA block copolymer matrix. The procedure was described in detail by Hong et al. [17] Briefly, the composite was composed of 60 wt% of the synthesized PGA–PLA block copolymers, 25 wt% of PGA fiber, and 15 wt% of HA-g-PLA. The synthesized PGA–PLA block copolymers and HA-g-PLA were vigorously mixed. The HA-g-PLA/HFIP (10 wt%) suspension was dispersed via sonication for 60 min; the PGA–PLA block copolymers/HFIP (5 wt%) solutions were generated with stirring. The above-mentioned mixture was precipitated in excess ethanol before being dried at 50 °C for 24 h in a vacuum oven. The mixture and PGA fiber were injection-molded (Bautek, Mini Molder BA-915A, Pocheon, Korea) at processing temperatures between 120 and 140 °C to generate the respective composite. The tensile strength of the samples was measured using the blended composites via the universal testing machine (UTM, LR10K Lloyds Instruments Ltd., West Sussex, UK). The composite samples for the in vivo degradation experiment were manufactured using a hot-press machine (Cat. 3925, Carver Inc., Wabash, IN, USA) at 120–140 °C to produce specimens that were 1 mm thick.

#### 2.2.4. Biological Responses by In Vitro Cytotoxicity Tests

The cytotoxicity MTT assay of the synthesized block copolymers and their composite samples were conducted to determine the associated cellular responses. MTT assay was conducted using a 3-(4,5-dimethylthiazolyl 2)-2,5-diphenyltetrazolium bromide (MTT)/phosphate-buffered saline (PBS) (5 mg/mL) solution in accordance with the ISO 10993-5 guidelines. Four block copolymer/composite samples were immersed in a growth medium containing HOb (4 g per 20 mL) and incubated at 37 °C for 24 h for exudation. The extracted medium was diluted to predetermined concentrations and immediately used for analysis. The HOb was cultured in a CO_2_ incubator (SL-205C, Thermo Fisher Scientific, Waltham, MA, USA). HOb cell suspensions (1 × 10^4^ cells/well) were seeded in 96-well flat-bottomed tissue culture polystyrene (TCPS) dish plates. After a 24 h incubation period, the cell culture media in each well was replaced with the extracted medium and subjected to an additional 24 h of incubation. Next, the medium was substituted with 100 μL of fresh medium and 25 μL of the MTT solution at a concentration of 1 mg/mL. After an additional 4 h incubation period, the culture medium was removed via aspiration, and DMSO (100 μL) was placed in each well to dissolve the formazan crystals synthesized from the MTT solution. The absorbance of formazan at 570 nm was measured using an enzyme-linked immunosorbent assay (ELISA) reader (SpectraMaxM5, Molecular Devices, Co., Ltd., San Jose, CA, USA). The relative cell viability was evaluated and compared with the control TCPS sample.

#### 2.2.5. Biological Responses by In Vivo Animal Biosafety Tests

The in vivo animal study was reviewed and approved by the Institutional Animal Care and Use Committee (IACUC) of Sungkyunkwan University, School of Medicine (SUSM) (Approval No. SKKUIACUC 2017-11-09-3). The skin sensitization test was conducted on the PGA–PLA block copolymers and their composites by first extracting each sample using PBS (4 g per 20 mL) for 72 h at 37 °C. The extracted solutions were then injected into the intradermal tissue of Sprague Dawley rats’ back (SD-rat, *n* = 3, 8 weeks old, Orient-Bio Inc., Seongnam, Korea). The anesthesia process consisted of insufflation narcosis using isoflurane (0.5–2.0 %) with O_2_ gas via the animal anesthesia system Serial No. 11107 from NorVap International Ltd. (Nelson, UK). Each spot was observed by the naked eye after injection for periods of 24, 48, and 72 h, and scored according to the guidelines set out by the Organization for Economic Co-operation and Development (OECD) for testing chemicals (Table 1). After the 72h skin sensitization test was complete, the injected SD-rats were sacrificed using a CO_2_ chamber, and an incision was made in the skin of their back to observe the occurrence of intradermal intracutaneous reactions at the injection site.

#### 2.2.6. In Vivo Degradation Test and Histochemical Analysis

This animal study was reviewed and approved by the IACUC of SUSM (Approval No. SKKUIACUC 2018-07-12-3). The in vivo degradation behavior of the PGA–PLA block copolymers and their composites were determined by inserting the samples intradermally in the SD-rats’ (*n* = 3, 8 weeks old, Orient-Bio Inc., Seongnam, Korea) back. The inserted samples were manufactured using an injection molding instrument and hot-pressed to the dimensions 5 × 1 mm (i.e., radius × height). Transplantation was conducted under insufflation narcosis using isoflurane (0.5–2.0%) with O_2_ gas via the above-mentioned animal anesthesia system for the fixation process. After transplantation was complete, the incision sites were sutured using nonabsorbable EZ clip wound closures (Stoelting Co., Wood Dale, IL, USA). The rats were injected subcutaneously with 40 mL/kg of Metacam^®^ (Boehringer Ingelheim Co., Ingelheim am Rhein, Germany) once a day as part of a pain management regime. The rats were subsequently sacrificed after a 1-, 2-, 3-, 4-, 5-, or 6-month period, and the implanted samples along with the surrounding tissue were extracted. The extracted samples were gently washed using PBS solution and dried under a vacuum. The tissue samples derived from SD-rats that had been sacrificed after 1 and 5 months were extracted and subjected to preparation procedures in paraffin for histochemical analysis. The histochemical analysis was conducted using hematoxylin & eosin (H&E)-stained section species to detect the nuclei of the associated macrophages that were derived from a foreign body reaction; immune-stained images sing clusters of differentiation (i.e., (CD)-68 antibody, Thermo Fisher Scientific, Rockford, IL, USA) were also obtained. Digital images of the paraffinized tissue sections were examined using slide scanner scope (Leica Biosystems, Aperio ScanScope^®^ CS System, Wetzlar, Germany).

## 3. Results and Discussion

### 3.1. Synthesis of PGA–b-PLA, b-PLLGA, and Their Composites

**Figure 2:**
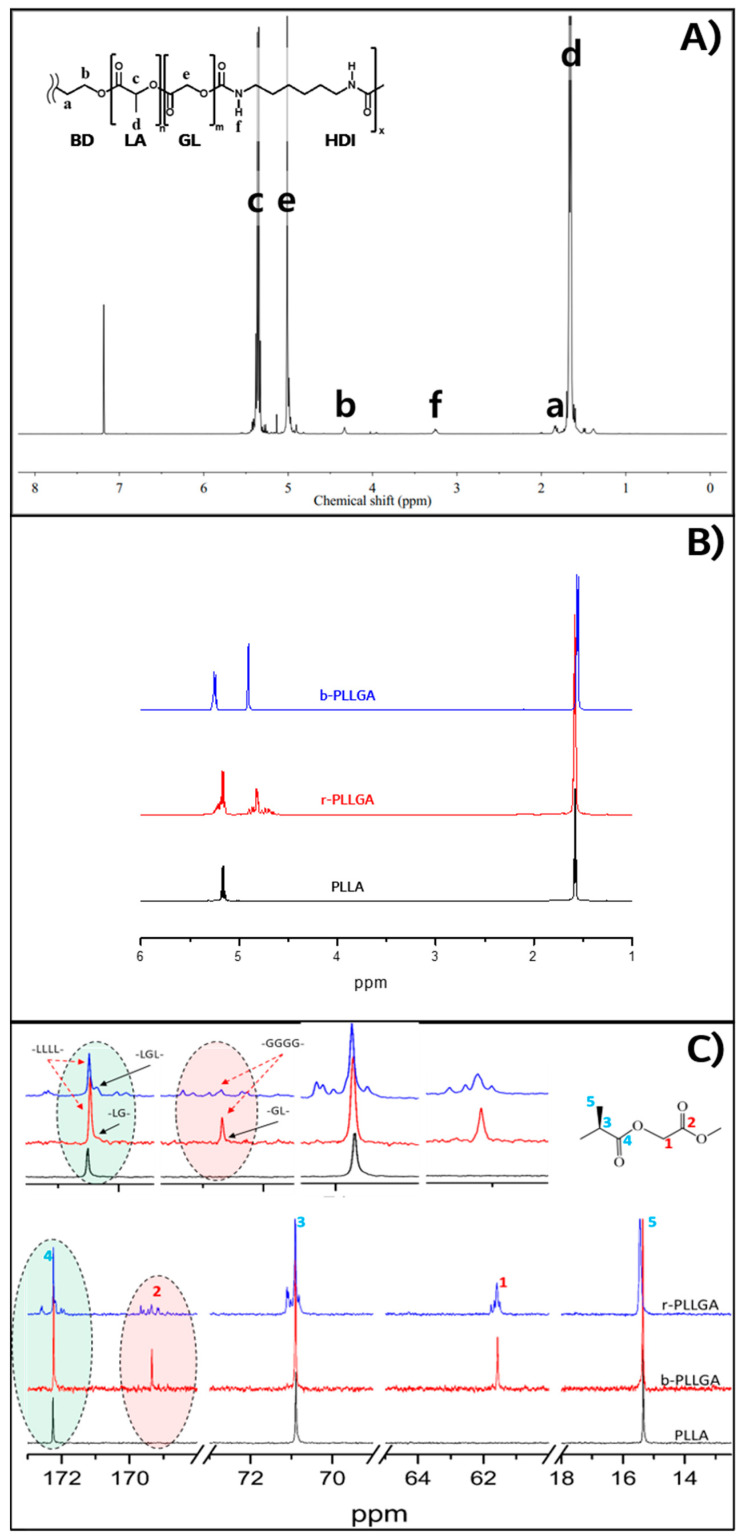
^1^H NMR spectrum of PGA–b-PLA (**A**), ^1^H NMR spectrum of b-PLLGA compared with the spectrum obtained from r-PLLGA and PLLA (**B**), ^13^CNMR spectrum of b-PLLGA compared with r-PLLGA and PLLA (**C**).

The structure of the synthesized PGA–b-PLA and b-PLLGA samples were characterized using nuclear magnetic resonance (NMR) spectroscopy. Here, ^1^H NMR spectra were recorded with a Bruker AVANCE III 400 instrument (Bruker BioSpin AG, Fällanden, Switzerland) using CF_3_COOD (trifluoroacetic acid-d) as the solvent. From the ^1^H NMR spectra shown in Figure 2A, we noted proton peaks that corresponded to the PLA and PGA block along with urethane bonds. The number of repeating units (*n_x_*) of the PLA and PGA block in the copolymer was determined from the ^1^H NMR spectrum by comparing the relative proton peak intensity (*x*) of an end-group with a known number of protons (*y*) to that of the repeating chain unit of interest using the following equation [18]:

(1) $$n_{x}=\frac{a_{x}m_{y}n_{y}}{a_{y}m_{x}}$$

where *a_x_* is the area or intensity of the ^1^H NMR peak associated with moiety *x*, *n_x_* is the number of repeating units of moiety *x*, *m_x_* is the number of protons of moiety *x*, *a_y_* is the area or intensity of the ^1^H NMR peak of moiety *y*, *n_y_* is the number of repeating units of moiety *y*, and *m_y_* is the number of protons of moiety *y*. The number of repeating units of the PLA block was calculated using the peak areas of the –CH_2_ (1,4-butanediol, δ 1.82) and –CH (PLA, δ 5.35) groups, whereas the number of repeating units of the PGA block was calculated using the peak areas of –CH (PLA, δ 5.35) and –CH_2_ (PGA, δ 5.02) (Table 2). The calculated *M_n_* values of the PLA and PGA blocks in the multiblock copolymer synthesized herein were 9400 and 1200, respectively. The PGA–b-PLA block copolymer was characterized via thermal transition analysis using differential scanning calorimetry (DSC, TA instruments DSC 2010, New Castle, DE, USA) at a heating rate of 10 °C/min with a temperature range from −20 to 250 °C under nitrogen. The first transition temperature (i.e., the T_m_ of the PLA block) was 154.3 °C, and the second transition temperature (i.e., the T_m_ of the PGA block) was 215.1 °C [19]. The heat of melting (ΔH_m_) was 38.2 and 13.3 J/g for the PLA and PGA blocks, respectively (data not shown). These results were concurrent with previously reported data [16].

The chemical structure of the random copolymers of poly(l-lactide-co-glycolide) (r-PLLGA), b-PLLGA, and homo-poly(L-lactide) (PLLA) were determined using deuterated trifluoroacetic acid and chloroform as the solvents. The ^1^H NMR and ^13^C NMR spectra were obtained using a Bruker NMR 400MHz (Bruker, Billerica, MA, USA). The molar ratio of glycolic acid and lactic acid in the feed and the ratio of the r-PLLGA and b-PLLGA were calculated from the ^1^H NMR spectra shown in Figure 2B. The ratio of glycolic acid in the copolymer was determined using the integrals of the area of the proton signals in the ^1^H NMR spectrum. The peaks observed at 1.60 ppm (r-PLLGA) and 1.68 ppm (b-PLLGA) were attributed to the –CH_3_ protons of the lactic acid component, whereas those noted between 4.6 ppm and 4.9 ppm (r-PLLGA) and at 5.1 ppm (b-PLLGA) were assigned to the –CH_2_ protons of the glycolic acid component. The peaks observed between 5.1 and 5.5 ppm (r-PLLGA) and at 5.4 ppm (b-PLLGA) were attributed to the –CH protons of the lactic acid units. Equation (2) below was used to calculate the molar percentage of the glycolic acid component in the copolymer:

(2) $$P_{GA}=\frac{I_{G}}{2I_{L}+I_{G}}\times 100$$

**Figure 3:**
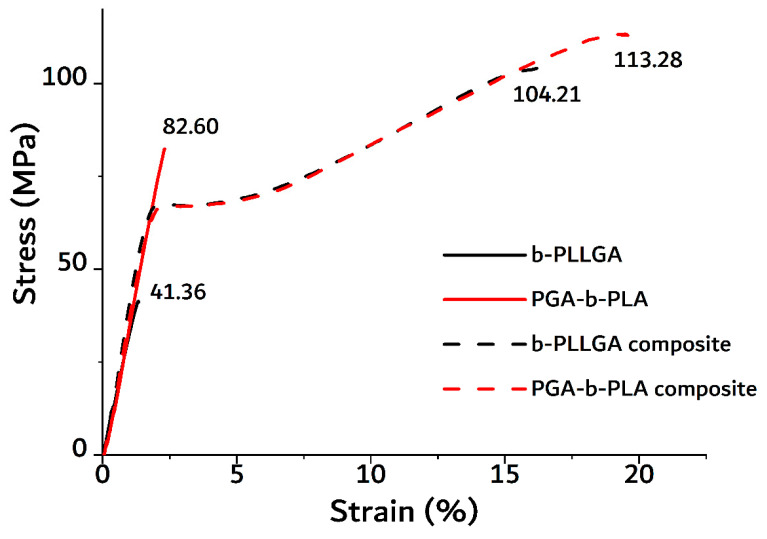
Tensile stress-strain curves of the PGA–b-PLA, b-PLLGA, PGA–b-PLA composite, and b-PLLGA composite.

**Figure 4:**
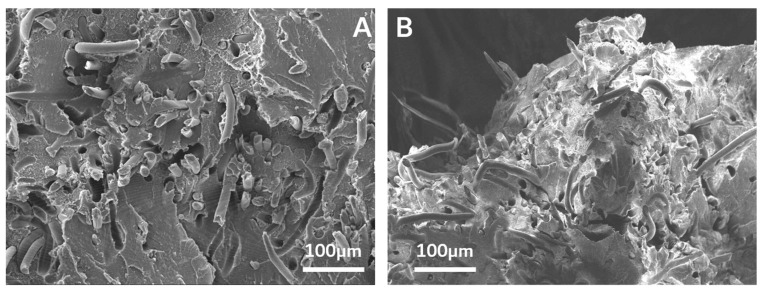
FE–SEM image of the breakage site of the PGA–PLA block copolymer composites for the PGA–b-PLA (**A**) and the b-PLLGA composites (**B**).

In the above equation, *P_GA_* represents the percentage content of the glycolic acid unit in b-PLLGA, *I_G_* is the integral of –CH_2_, and *I_L_* is the integral of –CH_3_. The percentages of glycolic acid determined from the ^1^H NMR spectra of the copolymers were 30.3% and 29.4% for r-PLLGA and b-PLLGA, respectively. These results of the glycolic acid and lactic acid ratios in the copolymers were concurrent with the target content stated in previous reports, which provided evidence of the successful synthesis of r-PLLGA and b-PLLGA [20]. The ^13^C NMR spectrum shown in Figure 2C highlighted the extreme sensitivity of the carbonyl carbons to their surroundings. The spectrum of PLLA homopolymer exhibited only three peaks in the carbonyl, methane, and methyl regions at 169.4, 70.9, and 15.4 ppm, respectively. Here, two new peaks appeared at 169.2 and 61.4 ppm with the addition of glycolic acid (b-PLLGA), and the resonance signals were attributed to the homo-sequences centered on the carbonyl region of the glycolidyl (−GGGG– at 169.2 ppm) and the methylene (−GGGG– at 61.4 ppm) regions. The peak intensity of the glycolidyl moiety increased at a higher glycolic acid feed ratio [16]. The PGA–PLA block copolymers were only soluble in HFIP. So inherent viscosity measurement was adapted as an available tool for molecular weight confirmation instead of GPC measurement. The solvent of inherent viscosity was HFIP, the concentration was 0.5 g/dL, and the temperature was 30 °C. The [η] of b-PLLGA and PGA–b-PLA were 0.43 and 0.9486 dL/g, respectively. For reference, the [η] of homo-PLLA (weight average molecular weight of 1.5 × 10^6^ g/mol, RESOMER^®^ LR 708, Evonik Industries, Essen, Germany) was 2.28 dL/g in the same condition. The data in Table 3 highlights the tensile strength of the synthesized block copolymers (PGA–b-PLA and b-PLLGA), which drastically increased after the HA–g-PLA filler and PGA fiber components were increased (i.e., b-PLLGA: 41.365 to 104.21 MPa and PGA–b-PLA: 82.605 to 113.28 MPa); all tensile strength tests were conducted according to the ISO 527-2 guidelines. And the raw data curves were shown in Figure 3. The above-mentioned composites exhibited remarkably improved tensile strength after the use of HA–g-PLA fillers, PGA fibers, and a matrix capable of consuming and elongating the fracture energy rather than facilitating the initial dewetting and cavitation processes taking place at the filler/matrix interface [17]. The cross-sectional areas of the composite samples were observed via SEM (JSM7000F, Jeol Co., Ltd., Tokyo, Japan); here, entangled HA–g-PLA fillers and PGA fibers were observed at the cross-sectional site of the composite samples (Figure 4).

### 3.2. In Vitro Cytotoxicity Test

**Figure 5:**
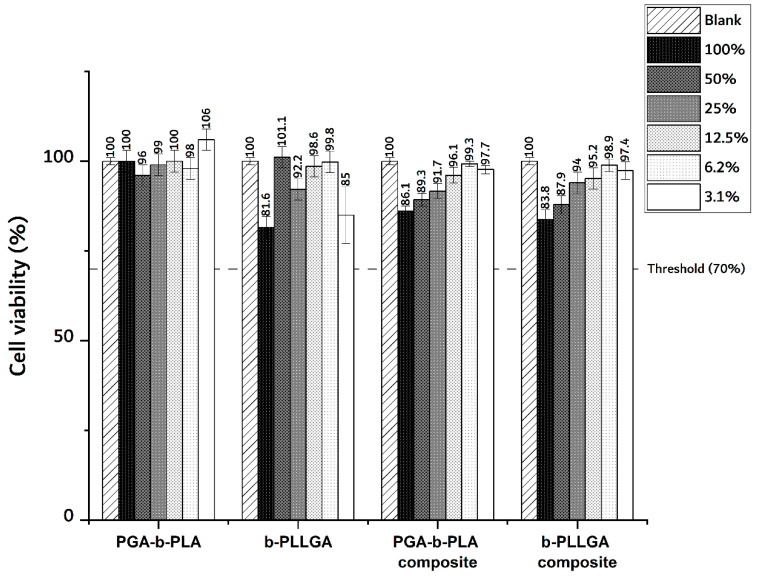
MTT assay of the PGA–b-PLA, b-PLLGA, the PGA–b-PLA composite, and the b-PLLGA composite (sample eluted in DMEM, *n* = 4).

The cytotoxic property of the produced samples was tested according to the ISO 10993-5 guidelines via MTT assay. The living cell transfer process from MTT tetrazolium to MTT formazan was conducted via the reduction of mitochondrial dehydrogenase. Here, MTT formazan was immiscible in aqueous solution and was subsequently precipitated from the MTT solution. The MTT formazan in the DMSO solution was measured at 570 nm, and this absorbance was proportional to the amount of MTT formazan formed; this was representative of the sample’s relative cell viability. The results of the MTT assay are summarized in Figure 5. From the MTT assay data, we noted that all samples exhibited non-cytotoxicity that was concurrent with ISO guidelines; thus, a 70% cell viability was considered as non-toxic [21]. These results indicated that the PGA–PLA block copolymers and their associated composites were non-cytotoxic even after being blended with the PGA fiber and HA–g-PLA filler. These results were useful for the next series of experiments (i.e., in vivo animal biosafety studies).

### 3.3. In Vivo Animal Biosafety Tests

**Figure 6:**
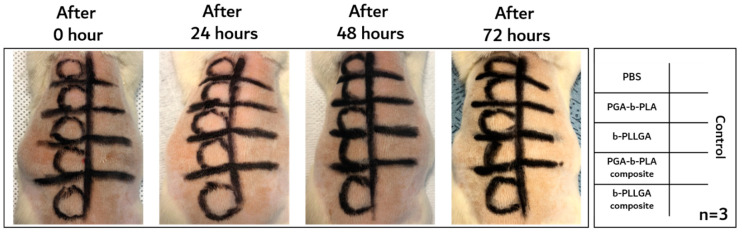
Skin sensitization analysis of the extracted solution injection obtained 24, 48, and 72 h after injection (*n* = 3).

**Figure 7:**
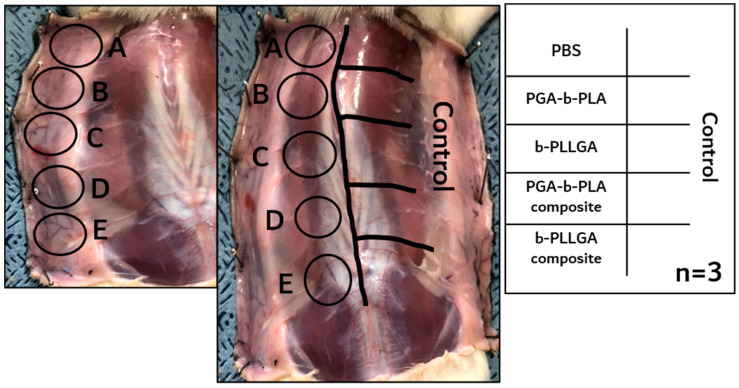
Intradermal intracutaneous reaction analysis of the extracted solution injection obtained 72 h after injection (*n* = 3) using the samples PBS (**A**), PGA–b-PLA (**B**), b-PLLGA (**C**), the PGA–b-PLA composite (**D**), and the b-PLLGA composite (**E**).

In vivo animal experiment for evaluating skin sensitization was tested according to the OECD guidelines listed in Table 1. As shown in Figure 6, all of the tested SD-rats exhibited a score of 0 after the 72h experiment after the extracts were injected (*n* = 3, Grade I). If the eluate caused sensitization, the skin turned red or exhibited swelling. The eluate-injected SD-rats that did not show any symptoms until 72 h were not sacrificed; instead, the skin at the incision site was debulked to observe the intracutaneous reaction. Generally, the formation of tumors and abscesses were typical reactions classified as a negative reaction. As shown in Figure 7, the debulked sites obtained from the sacrificed SD-rats also showed no negative reaction at the intradermal site (*n* = 3). These results meant that the extracted eluate of the samples induced no negative response during the biosafety and skin sensitization tests [22].

### 3.4. In Vivo Degradation Test and Histochemical Analysis

**Figure 8:**
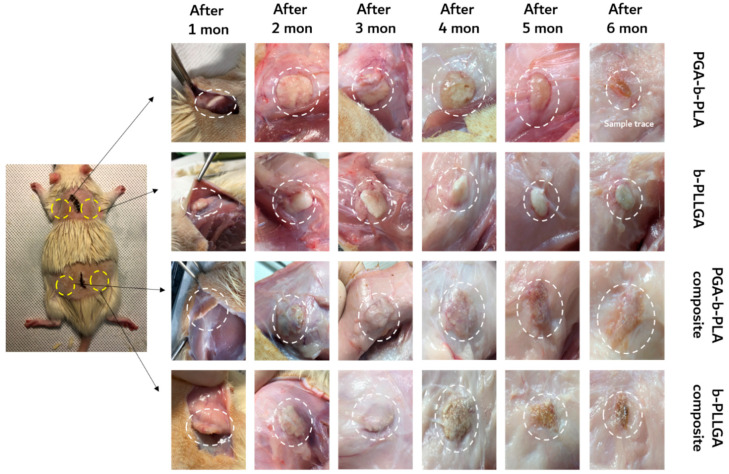
Images of the inserted in vivo animal degradation test samples (interval of extraction: 1 month, SD-rat, *n* = 3).

**Figure 9:**
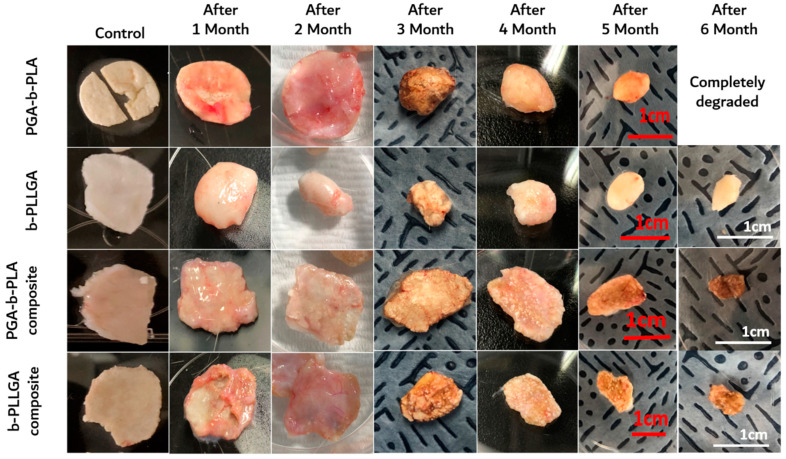
Images of the extracted in vivo animal degradation test samples (interval of extraction: 1 month, SD-rat, *n* = 3).

The animal studies were reviewed and approved by the IACUC of SUSM (Approval No. SKKUIACUC 2018-07-12-3). As shown in Figure 8, the implanted samples at the insertion sites (i.e., in the back of the sacrificed SD-rats) was incised according to a predetermined schedule, and the surface morphological changes of the samples were observed with a digital camera. All inserted samples exhibited swelling and surface degradation during the implantation period. Digital images of the removed samples were obtained for the 1- to 6-month SD-rat samples (Figure 9). Two months after insertion, we noted that the samples maintained their original shape; however, the recovered samples showed size and shape changes three months after insertion. The PGA–b-PLA completely disappeared via degradation in the SD-rats six months after surgery.

**Figure 10:**
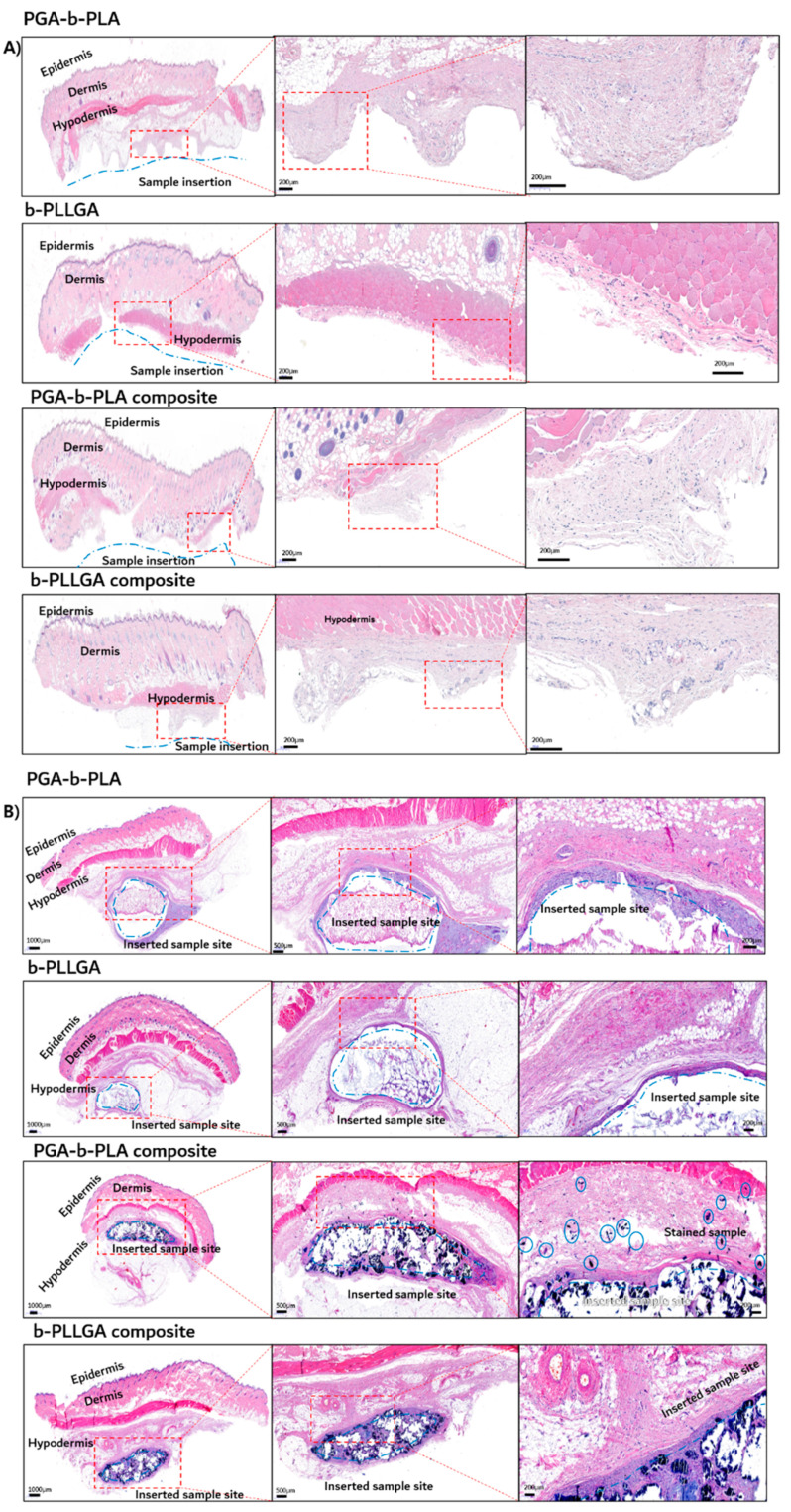
Histochemical analysis of the in vivo animal degradation test using H&E staining with insertion after 1 month (**A**) and H&E staining with insertion after 5 months (**B**).

**Figure 11:**
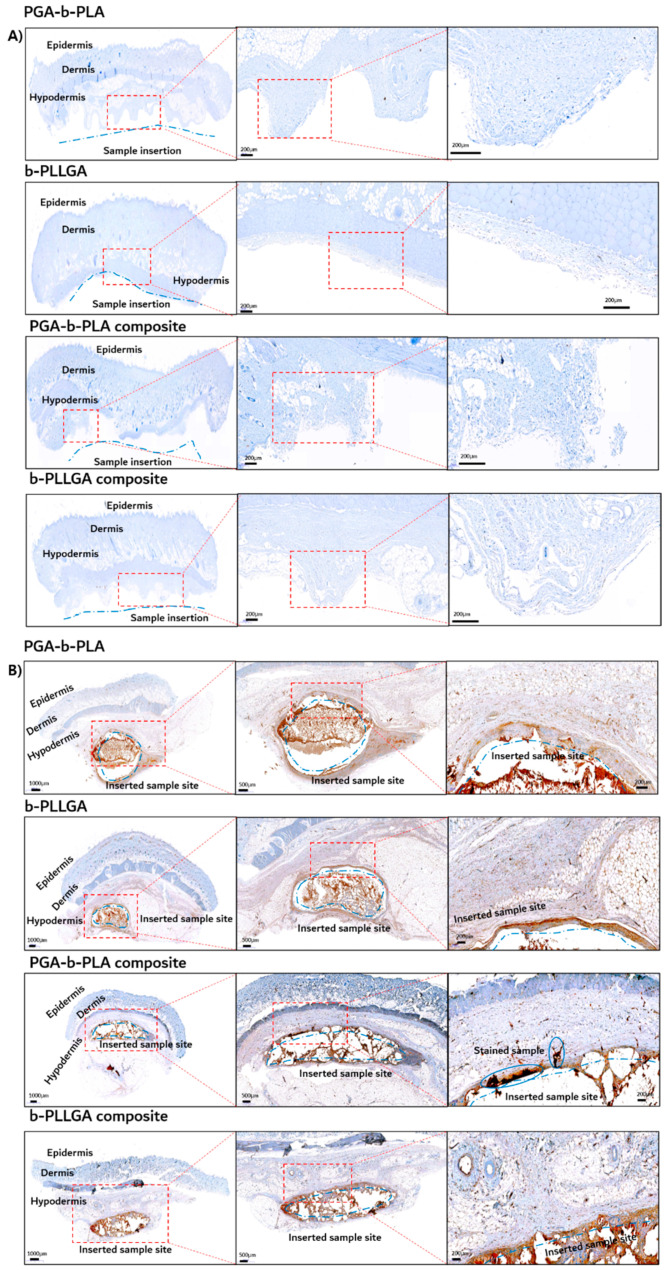
Histochemical analysis of the in vivo animal degradation test using immune staining with insertion after 1 month (**A**), immune staining with insertion after 5 months (**B**) (antibody: CD-68).

The tissues around the 1- and 5-month inserted samples were extracted and fixed using 10% neutral buffered formalin (NBF) for histochemical analysis. Next, the paraffin-embedded samples were stained with H&E, followed by CD-68, to detect the nuclei of the macrophages. The H&E-stained tissues are shown in Figure 10, and the immune-stained tissues using CD-68 are shown in Figure 11. Inflammation was not observed in the 1-month sample (Figure 10A and Figure 11A), which indicated that the inserted sample did not trigger any immunological irritation. In the 5-month sample (Figure 10B and Figure 11B), irritation was noted in the granuloma, but none was observed in the involved cells or giant cells. No inflammation was observed in the biopsies of the samples, and these samples exhibited good biocompatibility (as noted in the in vivo degradation test) [22,23].

## 4. Conclusions

In this study, synthesized PGA–PLA block copolymers and PGA–PLA block copolymer composites exhibited good biocompatibility. From in vitro MTT assay, we noted that the samples were non-toxic per ISO guidelines. In vivo animal biosafety tests revealed that the samples triggered no negative responses in the skin and at the intradermal site. The in vivo degradation test and histochemical analysis revealed that the samples were degradable after a 6-month implantation period with few signs of irritation via degraded debris. From these results, the PGA–PLA block copolymers and their composites were good candidates for degradable spinal fixation material.

## Figures and Tables

**Table 1:** Magnussin and Kilgman test, standard classes of Organization for Economic Co-operation and Development (OECD) guidelines for testing chemicals No. 406.

Score for Skin Sensitization Test (Magnussin and Kilgman)		Grade for Skin Sensitization Test (Magnussin and Kilgman)		
Standard	Score	Sensitization Rate (%)	Grade	Class
No reaction	0	0~8	I	Weak
Scattered mild redness	1	9~28	II	Mild
Moderate and diffuse redness	2	29~64	III	Moderate
Intense redness and swelling	3	65~80	IV	Strong
		81~100	V	Extreme

**Table 2:** ^1^H NMR data of PGA–b-PLA multiblock copolymer.

Position		δ (ppm)	Multiplicity	Peak Area
a	–CH_2_ of 1,4-butanediol	1.82	multiplet	0.32
b	–CH_2_ of 1,4-butanediol	4.34	multiplet	0.32
c	–CH of PLA	5.35	quartet	0.96
d	–CH_3_ of PLA	1.66	doublet	3.0
e	–CH_2_ of PGA	5.02	singlet	0.59
f	–NHCOO– of the urethane bond	3.23	quartet	0.32

**Table 3:** Tensile test results of UTM according to the ISO 527-2 guideline.

Sample Name	Tensile Strength (MPa)
PGA–b-PLA	82.605
b-PLLGA	41.365
PGA–b-PLA composite	113.28
b-PLLGA composite	104.21
